# Shooting from the ‘Scrip: Scope of Practice Laws and Access to Immunizations in the Pharmacy Setting

**DOI:** 10.3390/vaccines9050444

**Published:** 2021-05-02

**Authors:** Charles Stoecker

**Affiliations:** Department of Global Health Management and Policy, School of Public Health and Tropical Medicine, Tulane University, New Orleans, LA 70112, USA; cfstoecker@tulane.edu

**Keywords:** scope of practice law, occupational licensing, regulation, immunization, influenza

## Abstract

In the past two decades, most states in the United States have added authorization for pharmacists to administer some vaccinations. Expansions of this authority have also come with prescription requirements or other regulatory burdens. The objective of this study was to evaluate the impact of these expansions on influenza immunization rates in adults age 65 and over. A panel data, differences-in-differences regression framework to control for state-level unobserved confounders and shocks at the national level was used on a combination of a dataset of state-level statute and regulatory changes and influenza immunization data from the Behavioral Risk Factor Surveillance System. Giving pharmacists permission to vaccinate had a positive impact on adult influenza immunization rates of 1.4 percentage points for adults age 65 and over. This effect was diminished by the presence of laws requiring pharmacists to obtain patient-specific prescriptions. There was no evidence that allowing pharmacists to administer vaccinations led patients to have fewer annual check-ups with physicians or not have a usual source of health care. Expanding pharmacists’ scope of practice laws to include administering the influenza vaccine had a positive impact on influenza shot uptake. This may have implications for relaxing restrictions on other forms of care that could be provided by pharmacists.

## 1. Introduction

On 19 August 2020, Alex Azar, Head of the United States Department of Health and Human Services, used emergency powers to allow pharmacists to administer vaccinations to children over 3 years of age. This directive overrode many state licensing laws that previously prohibited pharmacists from administering some vaccines to some patients.

States in the United States each have separate authority to regulate the scope of medical licenses for pharmacists. Over the past six decades, most states have given additional authority to pharmacists to administer vaccinations to adults. These authorities may take several forms, ranging from the authority to prescribe and administer vaccines to only being allowed to administer vaccines when the patient had already been prescribed the vaccine by a licensed medical doctor. The objective of this study was to examine the impact of expanded scope of practice for pharmacists in the United States on influenza vaccine uptake rates in those age 65+.

There are some limited studies that have examined the potential impact of these channels for pharmacists to increase immunization rates. Early work has found causal evidence that pharmacists could increase immunization rates through mailed reminders [1], even when not administering immunizations directly. Cost-effectiveness modeling predicted that these reminders and related verbal cues would be cost-saving from the payer perspective [2]. These cost savings resulted from harnessing pharmacists’ messaging power to increase immunization rates among vulnerable populations. Pharmacists also have a potentially important impact on access to care through geographic proximity between health facilities and patients seeking preventative care [3]. With 62,000 pharmacy locations as of 2015 [4], pharmacies are potentially well-positioned to provide convenient access to preventative services such as immunization. An experiment in rural West Virginia found that parents who immunized their children in pharmacies cited the additional operating hours and more convenient locations as why they immunized their children at the pharmacy rather than a traditional physician’s clinic. As immunizations were not within pharmacists’ legislated scope of practice during the time of the experiment, nurses were brought into each pharmacy to administer the immunization [5]. Expanding the scope of practice for pharmacists to administer vaccinations themselves has increased the potential for replicating the West Virginia experiment nationwide. This expanded access increases the convenience of vaccinations for people previously vaccinated, but also has the potential to reach previously unvaccinated populations. In a survey of people vaccinated for influenza in the pharmacy setting, 25.5% were found to have been unvaccinated in the previous year [6]. The combination of advertising, lower cost, and increased convenience provided by allowing pharmacists to administer vaccinations is a potentially significant source for increased immunization rates.

In addition to potential increased vaccine access, broadening pharmacists’ authorized scope of practice may move care from high-cost to low-cost settings. When scope of practice laws are set restrictively enough to exclude services that can be safely done by that profession, prices of care increase. By allowing pharmacists to administer vaccines, we may decrease the overall cost of vaccinations by moving them from expensive clinics into lower-cost pharmacies. This study examines of effects of expanding pharmacists’ scope of practice to include immunization on influenza immunization rates.

## 2. Materials and Methods

Data on the primary outcome measure, whether or not an individual was immunized, came from the Behavioral Risk Factor Surveillance System (BRFSS) [7] from 1993 to 2014. The BRFSS is an annual, cross-sectional, nationally representative telephone survey of adults aged 18 years and older. Beginning in 1993, the BRFSS asked respondents to report if they had received a flu shot during the past 12 months. Starting with the 2004 survey, respondents were also asked if they had received the flu nasal spray. In 2011, these questions were combined, asking respondents if they had received either the nasal spray or the flu shot. To make outcomes more comparable over time, this study considered a respondent immunized if they responded yes to receiving either the nasal spray or the injectable immunization.

In selected years and states, respondents who indicated receipt of flu immunization were asked whether that immunization was in a store-based setting (drug store or grocery store) or elsewhere. Respondents in all states and years were asked whether they had had a check-up in a doctor’s office in the last 12 months and whether they had a usual place to access health care.

The Advisory Committee on Immunization Practices (ACIP) makes recommendations about which vaccines to administer and at which ages these vaccines should be given. The 65 and older population was recommended to receive the influenza vaccine before the BRFSS started asking respondents about their vaccination status (1993). Over time, more adults have been recommended to be vaccinated: adults age 50–64 in 2000 [8] and adults 19–49 in 2010 [9]. For this reason, the analysis focused on adults age 65 and older as they were recommended to receive the vaccine throughout the years that vaccination status is observed in the BRFSS [10].

**Figure 1:**
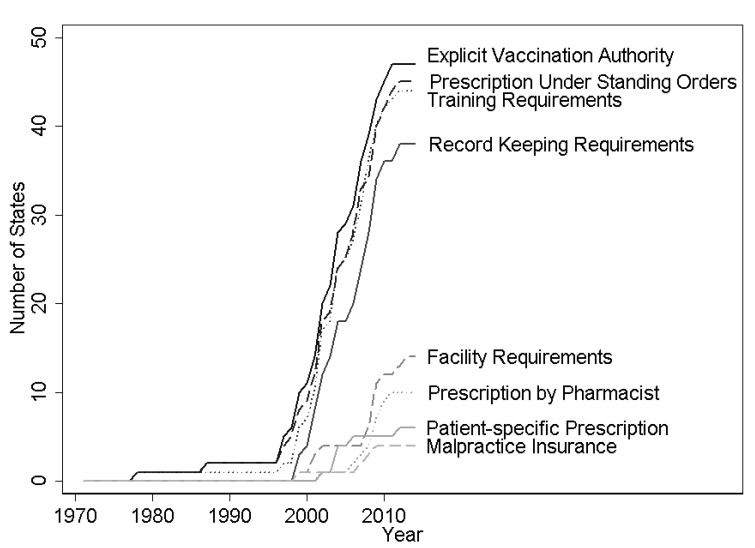
Expansions and limitations on pharmacists’ vaccination authority, 1971–2014. Note: Figure shows count of states in each year that give pharmacists the indicated vaccination authority or impose the indicated requirements for pharmacists to administer vaccines. Data from WestLaw Classic and WestLawNext legal databases.

Data on regulations on pharmacists’ authorization to conduct immunizations were collected by the Centers for Disease Control and Prevention’s Public Health Law Program using the WestLaw Classic (Thomson Reuters, Toronto, ON, Canada) and WestLawNext legal databases (Thomson Reuters, Toronto, ON, Canada). The effective date was recorded for each change in statute or regulation for pharmacists’ practice of immunization. These authorizations can be broadly categorized into (1) authorization for administration, (2) authorization for prescription, and (3) regulations on practice. In category (1), scope of practice expansion was dated from when pharmacists were explicitly authorized to administer vaccinations. This important permission was analyzed alone and then it was controlled for when considering the impacts of modifiers in categories (2) and (3). Category (2), authorization for prescriptions, falls into three possible levels. The most restrictive set of permissions required a patient-specific prescription from a doctor before the pharmacist could administer immunization. A more permissive regime required the pharmacist to obtain a standing order from a doctor that indicated which types of patients could be vaccinated at the pharmacy. A standing order might indicate that the pharmacist should seek to vaccinate any unvaccinated individual that was not contraindicated to receive the influenza vaccine due to underlying health issues and might last for the current influenza season. The most permissive regime gave the pharmacist authority to prescribe the influenza vaccination without involving a doctor. Category (3), regulations on practice, pertained to requirements for the pharmacist to receive extra training or certification, distribute a Vaccine Information Statement (VIS), keep records of the immunization or report those immunizations, maintain certain facility characteristics (e.g., maintaining patient privacy and an aseptic environment), or carry malpractice insurance. The evolution of state regulations on these aspects of pharmacists’ scope is shown in Figure 1. States generally regulated several aspects of pharmacists’ practice of immunization in the first year that pharmacists are given explicit authorization to vaccinate, and further regulation is infrequent (Appendix A Table A3). The earliest state recorded to pass regulation on pharmacists and immunizations was New York in 1971 and states continued to modify pharmacists’ authority and restrictions through the end of the legal dataset (2014).

To estimate the impact of authorizing pharmacists to administer the influenza vaccine on influenza immunization rates, this study used a differences-in-differences model with state and year fixed effects. Regressions controlled for individual-level characteristics including age in years, indicators for being white, black, Hispanic, unemployed, married, or widowed, having no insurance, and having less than a high school degree, a high school degree, some college, or graduating from college. All standard errors were clustered at the state level to account for the serial correlation in regulations on pharmacists within a state over time. Similar specifications were used to examine the impact of pharmacists’ authority on the probability of receiving a flu shot in a store (conditional on receiving a flu shot), the probability of having had a check-up in a doctor’s office in the last 12 months, and whether the individual had a usual place to access care.

As a robustness check, and to control for possibly spurious correlations between states relaxing regulations on pharmacists’ scope of practice laws and trends in vaccinations, models that included state-specific linear and quadratic time trends were also estimated. An event study analysis was conducted as a further robustness check [11], which examined the leads and lags of the policy variable. For the event study, the sample was restricted to individuals in states with at least four years of BRFSS data before and after the policy change. All statistical analyses used StataMP Version 15.1 [12] (StataCorp, College Station, TX, USA).

## 3. Results

Summary statistics from BRFSS data show that influenza immunization rates were higher after this authorization was given (Table 1), but this simple comparison does not account for national trends and state-level differences.

Granting pharmacists explicit authority to administer vaccines increased the influenza immunization rate for those aged 65 and older by 1.4 percentage points (Table 2, column 2). This result is robust to the inclusion of state-specific linear and quadratic time trends.

**Figure 2:**
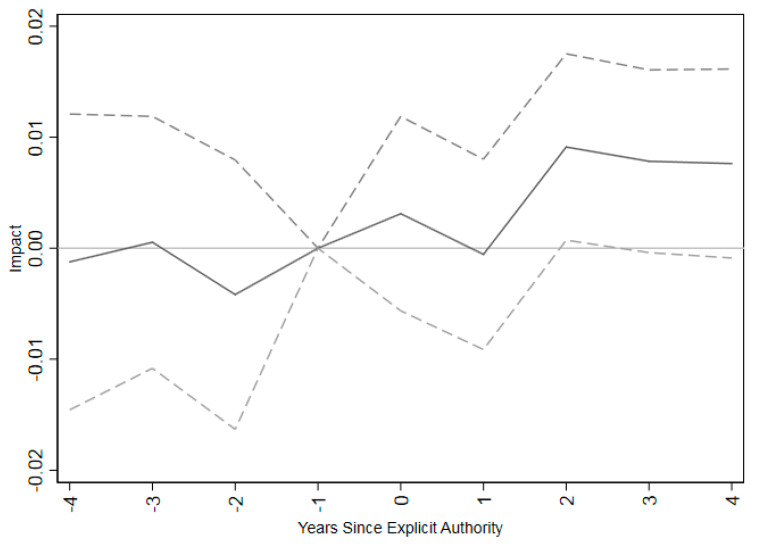
Event study for impact of pharmacists’ explicit authorization to vaccinate on influenza vaccination coverage rates for adults age 65+. Note: The figure shows coefficients (connected by solid lines) and 95% confidence intervals (connected by dashed lines) for a regression of influenza vaccination coverage rates on time relative to implementing explicit permission for pharmacists to administer vaccinations. The omitted category is one year prior to vaccination. The regression includes only states that have at least 4 years of vaccination data before and after granting explicit authority to administer vaccines for pharmacists. Regressions are at the individual level, weighted by appropriate survey weight from BRFSS data and clustered at the state level. Individual controls include dummies for income in bins, marital status, educational attainment, white, black, Hispanic, insurance status, and unemployment status as well as age as a continuous variable.

A key assumption of a differences-in-differences model is that trends in outcomes were parallel for the treatment and control groups in the absence of treatment. While this is not directly testable, suggestive support can be found by testing whether the pre-trends are parallel between the two groups. This was tested by looking at leads of the policy variable (e.g., were there differences in trends between the two groups in the pre-period?). If coefficients on the lead variables are statistically insignificant, it would indicate no association with the policy variable in the pre-period. There were no differences in pre-treatment trends in the four years preceding the introduction of explicit vaccine administration authorization for pharmacists (Figure 2). Immunization rates do not respond to scope of practice laws until two years after policy implementation.

If pharmacists were driving this increase in influenza vaccination, we would expect an increased share of influenza vaccines to be given in pharmacies. Column 1 of Table 3 provides some suggestive evidence that increased explicit authorization for pharmacists to administer vaccinations was associated with increases in vaccinations in the store setting. The data presented are for a linear probability model in a sample conditional on receiving an immunization. (The decision on where to receive a shot is likely to be part of the decision of whether to receive a shot at all. A multinomial logit model was estimated where the choices were to forego the flu shot (reference), receive a flu shot in any location but a pharmacy, or receive a flu shot in a pharmacy. This model is presented in Appendix A Table A2 and shows an association between states passing laws that allow vaccination in pharmacy settings and individuals getting a flu vaccine in a store. Further, it shows that allowing pharmacists to administer vaccinations is also linked to increases in vaccinations in clinics, possibly indicating spillovers from advertising campaigns by pharmacies. It is likely, however, that the choices to receive a shot in the pharmacy or another setting do not pass the test of independent irrelevant alternatives (IIA). A suest-based Hausman test of the IIA assumption reveals that it is violated (*p* < 0.001).) This analysis is subject to two important data limitations. First, the BRFSS did not expressly ask the respondent if they were vaccinated in a pharmacy explicitly, but rather if they were vaccinated in any store setting (including drug stores and supermarkets). Second, while the BRFSS has some data on shot receipt, they were only collected in selected years (1999, 2002, 2003, 2004, 2005, 2011, 2012, 2013, and 2014), and, further, not all states asked the question even in these selected years. Thus, the analysis in Table 3 was based on an unbalanced panel. (As balancing the panel is not feasible due to missing years in the middle of the sample, a test for non-random missing values [13] was run. The sample was limited to individuals that reported having the flu shot in all states, regardless of whether that state administered the question about location of receipt. Individuals that had a missing value for location of receipt were coded as 1 and those who responded to the question were coded as 0. This dummy variable was then used as an outcome variable in a differences-in-differences model. Results were statistically insignificant, indicating that missing data on location of receipt were random with respect to authorizing pharmacists to administer vaccinations.) Given these caveats, allowing pharmacists to administer vaccinations was associated with an increase in store-based immunizations of 1.4 percentage points for those aged 65 and older after conditioning on being immunized. This represented a 17 percent increase over a pre-explicit authorization baseline of 8 percent.

Spillover impacts were also examined with two access measures of care in a traditional setting from the BRFSS: whether the individual had a check-up in a doctor’s office in the last 12 months and whether the individual had a usual place to access health care. Authorizing immunizations in pharmacies had no clinically meaningful impact (<0.1%) on either of these measures (column 2 and column 3 in Table 3).

Getting vaccinated in a pharmacy is potentially easier if pharmacists can administer vaccinations under their own prescriptive authority. Requiring a patient-specific prescription before a pharmacist can administer a vaccine had a strong negative impact of −1.7 percentage points, which was large enough to cancel the gain from giving pharmacists the authority to administer vaccinations (Table 4). The impacts of other restrictions were statistically insignificant.

## 4. Discussion

Giving pharmacists explicit authority to administer vaccinations increased immunization rates among those aged 65 and over by 1.4 percentage points. This increase in immunization due to policy change was comparable in magnitude to other studies examining methods to increase immunization rates, such as using text-messaging to boost immunization rates for parents of children (4%) [14] or pregnant women (1.7%) [15]. While the effect found in this study is much smaller than those reported from randomized trials for offering immunizations in the workplace (14%) [16], it is also capable of reaching those not attached to the workplace and most at risk for influenza—those aged 65 and older.

The event study shows that the impacts of giving pharmacists explicit permission to perform vaccinations are not statistically significantly different from zero until two years after the law goes into effect. It may take pharmacies time to arrange for training and become comfortable enough to offer the new service. While training courses provided by the American Pharmacist’s Association are brief, it may take time to arrange attendance and enroll. It also may take time for advertisements for immunizations in pharmacies to change patients’ immunization habits.

My findings do differ from a previous national-level differences-in-differences study using similar data in the United States [17]. There, the authors found no statistically significant relationship when comparing states that permitted pharmacists to administer substances and adult influenza vaccination rates. Three key differences in approach explain the divergent findings. First, the authors perform a subgroup analysis looking solely at those aged 65+ and find marginally statistically significant impacts. Indeed, their 95% confidence interval for this subpopulation includes the point estimates presented here. This may be partially explained by my study using additional years of the BRFSS. Further, examining groups under age 65 is potentially problematic as their influenza recommendations changed over this time period. Second, my study examines the impact of explicit authorization. Many states authorized pharmacists to administer medications, including injections, before explicitly authorizing them to administer vaccinations. Technically, the more general authorization would include the authority to administer vaccinations, yet, like the prior studies, this study found no relationship between the more general authorization and immunization rates. It is only the explicit authorization that is associated with increased immunization rates. This may indicate that pharmacists are waiting for explicit permission before practicing to the fullest extent of their license. Third, in this study, the different modes of prescriptive authority were modeled as modifiers on the explicit authorization for vaccinations rather than considering a requirement for a prescription from a licensed physician as no permission. The findings in this study are, however, consistent with a recent meta-analysis of pharmacist immunization programs in specific localities [18] that found that pharmacists substantially increased immunization rates, and other, more recent, studies in Wales and Nova Scotia [19,20].

Pharmacists have larger impacts on influenza immunization rates when laws granting explicit authority to administer vaccinations are coupled with granting prescriptive authority to pharmacists for those vaccinations. Allowing pharmacists to be a one-stop shop for immunizations presents more convenience for the patient. This may make patients more likely to be immunized if the process is easier. Further, by allowing pharmacists to immunize patients on the spot, the pharmacists may more aggressively promote immunizations both in advertising through mailers and external signs as well as in-person when potential patients are in the pharmacies on business unrelated to immunization.

There are several important limitations to acknowledge in this study. First, as previously noted, immunization status was self-reported. While self-reported immunization status generally has a high sensitivity, it has a low specificity [21,22]. This is only a problem for this study if granting pharmacists authority for vaccinations is correlated with an increased recall in vaccination status. This may lead to overstating the true impact of these statutes and regulations if pharmacists’ advertising campaigns for immunization services cause an increase in this mistaken recall of a vaccination by the unvaccinated. Second, the BRFSS switched to a cell-phone and land-line telephone sampling frame from the previous land-line telephone-only frame starting in 2011. While the year fixed effects in the differences-in-differences model will control for any change in levels of immunization coverage, the change in sampling frame may undermine the parallel trends assumption. After limiting the sample to the land-line telephone-only sampling periods (1993–2010), the results were largely unaffected (Appendix A Table A1).

This study has narrowly focused on the impacts of expanding scope of practice regulations on pharmacists. As documented here, in jurisdictions where pharmacists’ scope of practice did not previously explicitly include administering vaccinations, including this explicit permission can increase immunization rates. However, there are limitations to the applicability of these findings to contexts outside of the United States. As has been previously noted, pharmacists may be reluctant to administer vaccines even if they are explicitly within their scope of practice without enough training. Further, if pharmacists are practicing in jurisdictions where there is no mechanism for reimbursement for vaccine administration, simply expanding the scope of practice is unlikely to achieve an impact on immunization rates [23].

## 5. Conclusions

Moving care from settings with high cost or inconvenience barriers to settings with lower barriers increases access to services including cost-effective preventative care. This study found increased utilization of preventative care in the form of influenza vaccination rates when one such low-barrier provider, a pharmacist, is explicitly authorized to administer influenza vaccinations. While many states authorize influenza vaccination for adults, prior to the emergency order from Secretary Azar, several states did not authorize pharmacists to administer other vaccines with lagging coverage rates, including HPV and Zoster. As physicians frequently do not provide a strong recommendation for receiving the HPV vaccine [24], pharmacists may be able to step into that gap. Consideration should be given to these authorities as that order expires. Removing barriers for these vaccines and other preventative services in alternative settings may have beneficial impacts on utilization rates.

## Figures and Tables

**Table 1:** Summary statistics from BRFSS data for adults age 65+.

	All Years		Before Authorization		After Authorization	
	Mean	SD	Mean	SD	Mean	SD
Had Flu Vaccination	63.7	(48.1)	62.2	(40.2)	64.8	(52.6)
Age	73.9	(6.5)	73.6	(5.2)	74.1	(7.5)
White	87.3	(33.3)	88.3	(26.1)	86.5	(38.8)
Black	8.3	(27.5)	8.6	(22.9)	8.0	(30.7)
Hispanic	5.8	(23.3)	4.0	(15.9)	7.2	(29.4)
No Insurance	2.2	(14.7)	2.3	(12.2)	2.1	(16.4)
Unemployed	90.5	(29.3)	91.1	(23.2)	90.0	(33.9)
Married	56.6	(49.6)	56.9	(40.3)	56.4	(56.1)
Widowed	29.2	(45.5)	30.8	(37.6)	27.9	(50.8)
Did Not Finish High School	19.6	(39.7)	23.2	(34.4)	16.6	(42.1)
High School Degree	33.6	(47.2)	34.2	(38.6)	33.1	(53.3)
Some College	23.1	(42.2)	21.3	(33.4)	24.6	(48.8)
College Gradate+	22.9	(42.0)	20.5	(32.9)	24.9	(49.0)
Check-Ups	86.2	(34.5)	85.7	(27.3)	86.6	(39.0)
Personal Doctor	93.9	(23.9)	93.4	(21.6)	94.2	(24.8)
In-Store | Any Vaccination	14.8	(35.5)	08.5	(24.5)	17.8	(40.3)

Note: Table shows means and standard deviations for BRFSS respondents from 1993 to 2014. Statistics are shown for all years (columns 1 and 2), observations from before each respondent’s state of residence passed explicit authorization for pharmacists to administer vaccinations (columns 3 and 4), and after such authorization (columns 5 and 6). Age is measured in years and all other variables are in percentages. “In-Store” is the percentage that received the flu shot in a store conditional on having the flu shot. Abbreviations: SD = standard deviation.

**Table 2:** Impact of explicit vaccination authority for pharmacists on influenza vaccine uptake rate for adults age 65+, 1993–2014.

	Model Includes State FE, Year FE	Model Includes State FE, Year FE, Individual Controls	Model Includes State FE, Year FE, Individual Controls, State Linear Time Trends	Model Includes State FE, Year FE, Individual Controls, State Linear Time Trends, State Quadratic Time Trends
Authorization	0.013 ***	0.014 ***	0.013 ***	0.013 ***
	(0.003)	(0.003)	(0.004)	(0.004)
Observations	1,654,657	1,258,825	1,258,825	1,258,825

Notes: Coefficients indicate the effect of state laws providing explicit authority for pharmacists to administer vaccinations on influenza vaccine uptake rate. Regressions are at the individual level, weighted by appropriate survey weight from BRFSS data and clustered at the state level. Individual controls include dummies for income in bins, marital status, educational attainment, white, black, Hispanic, insurance status, and unemployment status as well as age as a continuous variable. Standard errors shown in parenthesis. *** *p* < 0.01. Abbreviations: FE = fixed effects.

**Table 3:** Impact of explicit vaccination authority for pharmacists on likelihood of receiving influenza in a store, receiving a check-up or having a personal doctor.

	Vaccination in Store	Check-Up	Personal Doctor
Impact	0.014 ***	−0.001	0.001
Standard Error	(0.005)	(0.003)	(0.002)
Mean	0.08	0.86	0.94
% Impact	17	<1%	<1%
Obs	206,720	1,177,983	1,220,403

Notes: Coefficients indicate the effect of state laws providing explicit authority for pharmacists to administer vaccinations on likelihood of receiving the influenza vaccine in a store (e.g., supermarket or drug store) (column 1), having a check-up in the last 12 months (column 2), or having a personal doctor (column 3). For column 1, the sample is restricted to respondents that answered yes to receiving the influenza vaccine and includes each year that the BRFSS asked this question, 1999, 2002, 2003, 2004, 2005, 2011, 2012, 2013, and 2014, for any states that asked the question in that year. Regressions are at the individual level, weighted by appropriate survey weight from BRFSS data and clustered at the state level. Individual controls include dummies for income in bins, marital status, educational attainment, white, black, Hispanic, insurance status, and unemployment status as well as age as a continuous variable. Standard errors shown in parenthesis. *** *p* < 0.01. Abbreviations: FE = fixed effects.

**Table 4:** Impacts of types of prescriptive authority and other regulations for pharmacists on influenza vaccine uptake rate, 1993–2014.

	Coeff (SE)
Authorization	0.019 ***
	(0.005)
Standing Orders	−0.005
	(0.005)
Patient-Specific Requirement	−0.017 **
	(0.007)
Recording Keeping Requirement	0.009
	(0.006)
Training Requirement	0.01
	(0.007)
Facility Specifications	0.007
	(0.005)
Malpractice Insurance Requirement	0.001
	(0.006)
Obs	1,258,825

Notes: Coefficients indicate the effect of state laws providing explicit authorization for pharmacists to administer vaccines combined with the type of prescriptive authority accompanying that permission: under the pharmacist’s own authority (omitted), under standing orders, and with patient-specific prescriptions on influenza vaccine uptake rate. Regressions are at the individual level, weighted by appropriate survey weight from BRFSS data and clustered at the state level. Individual controls include dummies for income in bins, marital status, educational attainment, white, black, Hispanic, insurance status, and unemployment status as well as age as a continuous variable. Standard errors (SE) shown in parenthesis. *** *p* < 0.01, ** *p* < 0.05.

## Data Availability

Not applicable.

## Appendix A

**Table A1:** Impact of explicit vaccination authority for pharmacists on influenza vaccine uptake rate, 1993–2010.

	Age 65+
	(3)
Impact	0.013 ***
Standard Error	(0.004)
Observations	790,992
State FE, Year FE	yes
Individual Controls	yes

Notes: Table excludes data from the BRFSS cell phone sampling frame which began in 2011. Coefficients indicate the effect of state laws providing explicit authority for pharmacists to administer vaccinations on influenza vaccine uptake rate. Regressions are at the individual level, weighted by appropriate survey weight from BRFSS data and clustered at the state level. Individual controls include dummies for income in bins, marital status, educational attainment, white, black, Hispanic, insurance status, and unemployment status as well as age as a continuous variable. Standard errors shown in parenthesis. *** *p* < 0.01.

**Table A2:** Multinomial logit model of the impact of explicit vaccination authority for pharmacists on likelihood of receiving influenza vaccine by location.

Independent Category	Impact
	(1)
No Vaccination (reference case)	
Vaccination in Other Locations	0.157 ***
	(0.031)
Vaccination in a Store	0.320 ***
	(0.065)
Obs	354,201

Notes: Coefficients are from a multinomial logit model showing the association between states granting pharmacists explicit permission to vaccination and the outcome, where the outcome categories are whether an individual received no influenza vaccination (reference case), a vaccination in a location other than a store, or a vaccination in a store. Each year that the BRFSS asked this question was included: 1999, 2002, 2003, 2004, 2005, 2011, 2012, 2013, and 2014. States are only included if they elected to ask this question in the BRFSS survey. Regressions are at the individual level. Individual controls include dummies for income in bins, marital status, educational attainment, white, black, Hispanic, insurance status, and unemployment status as well as age as a continuous variable. Standard errors shown in parenthesis. *** *p* < 0.01.

**Table A3:** Elapsed time between explicit authorization and other regulations on pharmacists’ immunization practice.

Category of Legal Regulation	0	1	2	3+
Prescription Under Standing Orders	39	3	2	1
Patient-Specific Prescription	5	1	0	0
Prescription by Pharmacist	5	1	0	4
Facility Requirements	7	3	1	3
Malpractice Insurance	3	0	0	1
Training Requirements	35	5	1	3
Record Keeping Requirements	25	7	2	4

Notes: Table shows the number of states that have passed a particular regulation on pharmacists’ practice of immunization relative to the time since pharmacists were given explicit permission to vaccinate. Columns indicate elapsed time in years and cells indicate counts of states.
